# Optimizing NEURON Simulation Environment Using Remote Memory Access with Recursive Doubling on Distributed Memory Systems

**DOI:** 10.1155/2016/3676582

**Published:** 2016-06-20

**Authors:** Danish Shehzad, Zeki Bozkuş

**Affiliations:** Computer Engineering Department, Kadir Has University, 34083 Istanbul, Turkey

## Abstract

Increase in complexity of neuronal network models escalated the efforts to make NEURON simulation environment efficient. The computational neuroscientists divided the equations into subnets amongst multiple processors for achieving better hardware performance. On parallel machines for neuronal networks, interprocessor spikes exchange consumes large section of overall simulation time. In NEURON for communication between processors Message Passing Interface (MPI) is used. MPI_Allgather collective is exercised for spikes exchange after each interval across distributed memory systems. The increase in number of processors though results in achieving concurrency and better performance but it inversely affects MPI_Allgather which increases communication time between processors. This necessitates improving communication methodology to decrease the spikes exchange time over distributed memory systems. This work has improved MPI_Allgather method using Remote Memory Access (RMA) by moving two-sided communication to one-sided communication, and use of recursive doubling mechanism facilitates achieving efficient communication between the processors in precise steps. This approach enhanced communication concurrency and has improved overall runtime making NEURON more efficient for simulation of large neuronal network models.

## 1. Introduction

The brains complex computational behavior necessitated developing large neuronal computational models. Huge amount of data is integrated by models which work on simulation tools to study the information regarding brain computational processing. This enables neuroscientists to practically observe the computational behavior similar to brain and to carry out experiments along with fluctuating processes on simulating environment. As a consequence better understanding of brain functionality can be attained and diseases like epilepsy, Parkinson's disease, and so forth can be diagnosed and cured. There is wide range of simulators that have been developed for simulating neuronal behavior. NEST, NEOSIM, SPLIT, and NEURON are few of important simulators in practice today [1–5]. The advantages of assorted simulation environments are that each simulator has a broad range of potency and this miscellany contributes to better development and understanding of large neuronal models simulation processes. The valuable aspect of diverse simulation environment is its sundry nature and wide-ranging strengths enabling better understanding of computational behavior of neuronal networks. This diversity has also resulted in improving the simulating environments capability of computations unfolding the novel perspectives in overall computation and simulation technology. One of the key beneficial features is that fast and efficient architectures of computers can be achieved by the help of these computational simulation environments and neuronal models. These have the ability to provide parallel, speedy, and efficient processing.

NEURON has become a widely adopted simulation tool for building and analyzing neuronal models, using them for solving multifaceted neuronal computations [6]. The performance of NEURON simulator can be increased to support parallel environments by efficient utilization of machines. On large number of processors interprocessor spikes exchange consumes greater fraction of the total simulation time. The MPI_Allgather method is used in NEURON for spikes exchange after incorporating the cell equations for smallest amount specific time taken between spikes initialization and release. MPI_Allgather in MPI has feature of two-sided communication based on first gathering on every processor and sharing out among all other processors in the communication world. This requires both sender and receiver to participate in the communication process explicitly and requires extra synchronization among processes. MPI_Allgather is adopted in neuronal networks for collective communication, thus using MPI_Allgather for processors to gather spikes from each processor and broadcasting to all the nodes in its MPI communication world. To calculate this limitation testing was done on two network models, Parscalebush and Parbulbnet [7, 8], by increasing number of processors and modifying number of cells and tstop. The communication time relatively increases along with number of processors. These models were downloaded from the ModelDB repository (http://senselab.med.yale.edu/) and used parallel models from Netmod [9]. In this work Remote Memory Access (RMA) one-sided communication with recursive doubling is implemented and tested to achieve better performance, thus reducing the communication overhead in NEURON.

This paper is organized as follows: we begin with discussion on related work in Section 2. While Section 3 gives background knowledge of NEURON, MPI, and recursive doubling, in Section 4 parallel simulation in NEURON helps to understand need for optimization. Implementation details and NEURON optimization using RMA based on recursive doubling are justified in Section 5 along with experimental results in Section 6. The last section discusses our conclusions and future work.

## 2. Related Work

Many studies exemplify that distributing network architecture over multiple processors has features of fast processing of data. For example, the scaffold functioning in NEURON for parallel simulations and performance scaling can be obtained by testing the model [9]. As far as the simulations of large spike-coupled neuronal networks are concerned they make use of parallel models for efficient simulation on large computational clusters [10]. Many simulation environments have the capability of provisioning desired functionalities including NEST [11], pGENESIS [12], SPLIT [13], NCS [1], and C2 [14]. These simulation environments give various advantages like increase in simulation speed with increase in number of processors; the rate of communication is limited until each processor had very little work to do. Interprocessor spikes exchange is one of the most important factors to be considered in parallel network simulators.

A standard Message Passing Interface (MPI) is widely adopted by most of simulators and functions on use of the nonblocking point-to-point message passing utility. NEURON selects basic spike distribution method, which functions to distribute spikes among all processors [9]. The “Allgather” technique normally uses MPI Allgather and irregularly MPI Allgatherv when there are additional spikes to be sent that do not fit in the fixed size MPI_Allgather buffer [7]. The major objective is to get a baseline for future assessment with more advanced point-to-point routing methodologies. For instance, in NEST it was observed that Allgather give improved performance on their 96-core cluster using InfiniBand switch in comparison with Complete Pairwise Exchange algorithm [15, 16]. In terms of computational performance improvement NEURON simulator on parallel architectures can be enhanced by massive computational parallelization using GPGPUs. GPU simulator that can surpass CPU by 20 times was developed by Scorcioni [17]. Large scale neural simulators memory limitations on large clusters were elaborated in [18].

In the NBC library nonblocking extensions of collective calls have been developed, which was presented in MPI-2.1 [19]. It provides nonblocking collective operations on top of MPI two-sided point-to-point communication. Nonblocking collective though is an integral part of MPI-3.0, but implementation of MPI-3.0 in NEURON is still not available. Remote Memory Access can move two-sided communication to one-sided communication, thus allowing synchronization overhead reduction [20]. The proposal for neighborhood collectives was presented in [21]. For Blue Gene/P DCMF, active message passing library was presented in [22], and for MPI collective optimization Component Collective Message Interface was presented in [23]. The use of MPI_Allgather method increases the communication time along with increase in number of processors.

## 3. Background

### 3.1. NEURON Simulation Environment

NEURON is a powerful simulation environment for performing experiments on models of neurons or network of neurons [24]. It is a tool for constructing, managing, and exercising biologically realistic neuronal models. The NEURON was extended from single CPU to multiple CPUs to support complex computational models simulation. It can run parallel simulations on small clusters with 10–50 processors to large scale Blue Gene Super Computer with thousands of processors [9]. For communication neurons generate spikes and usually send to thousands of other neurons and receive from thousands of neurons. NEURON source cells and their target are usually not on the same processors; to handle this global identifier to cells on each processor they are assigned and messages are passed between hosts with appropriate weights and delays. Multiple processors use MPI_Allgather collective communication method to exchange spikes between processors [9].

### 3.2. MPI

In multiprocessing environment processors either perform multiple tasks simultaneously or distribute the same task across multiple processors for achieving the adequate level of concurrency. Message Passing Interface is used for communication between parallel processors running processes on distributed systems. MPI is an application programmer interface for inscription of message passing parallel programs which functions to cover the details of underlying system architecture. MPI is implemented as library since it enables convenient program that can manage to run similar program on parallel processors and has gone through enhancements in various versions [25–27]. Communication using MPI can be point-to-point or it can be collective communication [28]. The basic methodology of MPI is having multiple processes on distributed memory systems which communicate using message passing. The numbers of programs are always constant during the execution of program. Debugging an MPI program is hard as program cannot be distinguished into modules. Load balancing and collaborative communication is also limitation in MPI. It has its own programming structure and initializes with MPI_Init after that task is distributed among processors in its communication world and the parallel execution is finished with MPI_Finalize.

### 3.3. MPI Two-Sided Communication

**Figure 1:**
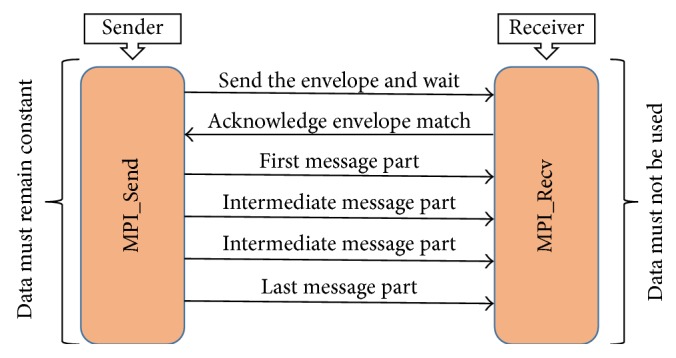
Two-sided communication in MPI.

MPI two-sided communication gives semantic assurance implied by the standard and its implementation is subject to various practical restraints. The communication pattern of MPI in two-sided communication is based on two subdivisions MPI_Send and MPI_Recv. MPI_Send routines are used for sending messages from source process to destination process and MPI_Recv routines for getting messages on target process sent by source process as shown in Figure 1. When processors are synchronized through acknowledgement of envelope match, data must remain constant during the communication process. The efficiency of program can be impacted by restrictions of synchronization in two-sided communication. Corresponding pattern of Send/Recv and message gathering limits choice for hardware message ordering as memory is private and sender has to wait until the receiver is ready to receive. Also support is required at recipient side for management of message size vagueness and message synchronization on both ends results in corresponding restriction for buffer allotment or memory registration. MPI_Send and MPI_Recv also restrict the communicating processors unless the complete data is transferred. There are two main communication mechanisms, firstly point-to-point one in which both sender and receiver participate explicitly and MPI collective communication which is used to optimize communication in wide area distributed environments [29, 30]. Different collective communication calls such as MPI_Gather, MPI_Allgather, and MPI_Reduce are used for communication among multiple processes.

### 3.4. MPI One-Sided Communication

**Figure 2:**
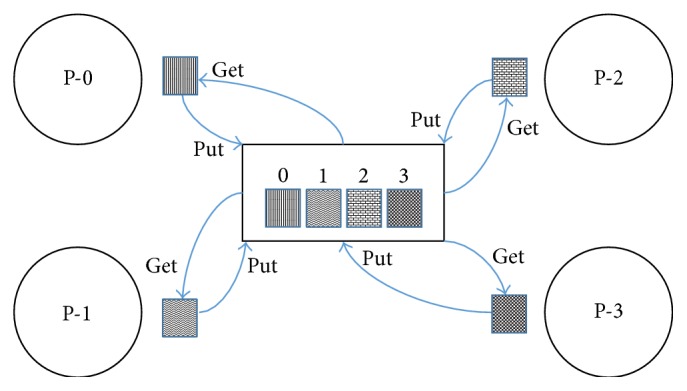
MPI one-sided communication.

All processes of MPI communication in MPI one-sided communication are carried out in framework of a window. Remote Memory Access unlike two-sided communication decouples data transfer from the synchronization of systems. Window is based on compilation of elements defined at the time of conception of window and adjacent area of memory at each process. One-sided communication can be used to send and receive data where one process can directly access memory address space of another process without intervention of other processes. Each processor declares a specified area of memory for remote access by MPI_Win_Create. The use of MPI-3 standard is based on three types of one-sided communication functions: MPI_Put, MPI_Get, and MPI_Accumulate. Sending data from source to destination on remote window is accomplished by using MPI_Put operation [31]. On the other hand MPI_Get is used to read data from the window of remote host as shown in Figure 2. MPI_Accumulate combines data into the target from the origin, thus becoming applicable by using MPI reduction operator which limits data into the buffer. The presence of communication functions takes place in framework of either active target synchronization time or passive target synchronization [32]. All communication processes are nonblocking and are finished without involving other processors to synchronize for communication and do not block both ends during the communication process.

### 3.5. Recursive Doubling Algorithm

**Figure 3:**
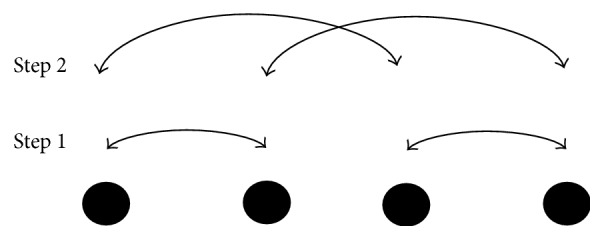
Recursive doubling mechanism for process communication.

The recursive doubling algorithm initially was developed to solve tridiagonal linear system of size *n* on a parallel computer with *p* processors using *O*(log⁡*p*) parallel arithmetic steps [33]. Recursive doubling mechanism can be used for collective communication between *p* processors requiring only *O*(log⁡*p*) number of steps. In each step processors communicate with other processors and distance among processors increases by power of 2 and size of message in each coming step doubles as compared to the previous step. Initially data exchange is carried out by the processes which are distance 1 apart from each other. After this, the processes which are distance 2 apart share the data received from previous step and their own data as shown in Figure 3. Thus data communication between processors is done in limited steps in efficient manner.

## 4. Parallel Simulation in NEURON

The collective communication in MPI resolves around the participation of all the processes in the communication group known as communication world. The synchronization of processes is mandatory; this means that all the processes in communication group reach the point of synchronization so they can continue execution. Spikes communication in NEURON is handled by MPI_Allgather by the collective process based on two-sided communication routine. MPI_Allgather is just a wrapper above MPI_Gather and MPI_Bcast, which in depth are another cover over MPI_Send and MPI_Recv for sending and receiving messages in the communication group.

Collective communication is the procedure of sending and receiving data amongst all the processors of MPI communication world. A processor in the MPI_Allgather communicator's world gathers data from every other process and distributes its own data amongst the communication group. In NEURON MPI_Allgather is used for communication between processors after each designated interval. The total runtime for simulation of models decreases when numbers of processors are increased.

**Figure 4:**
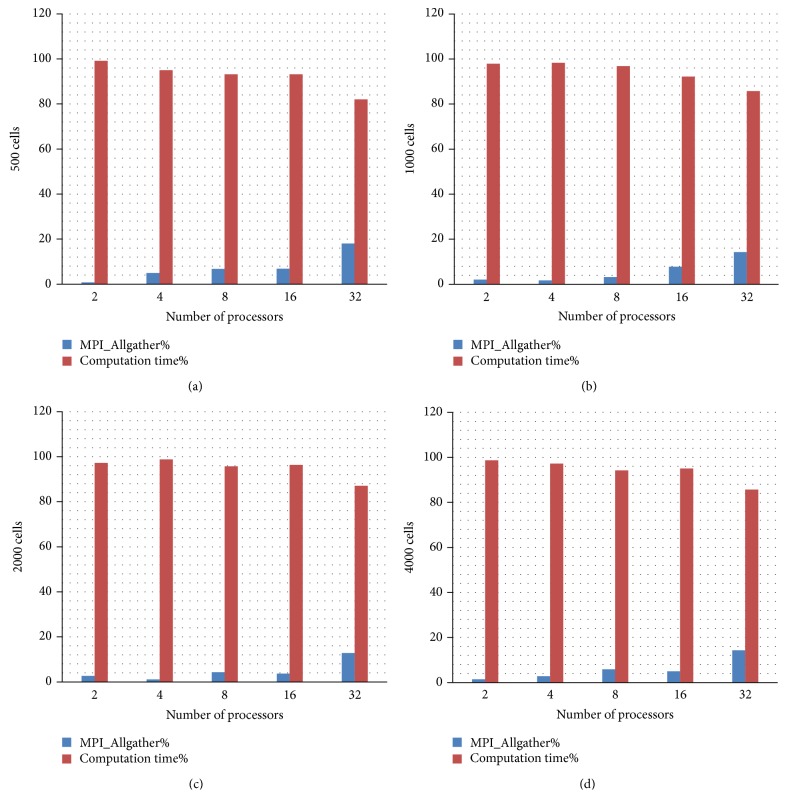
Parscalebush model spikes exchange time and computation time analysis graph.

On the other hand for communication between processors MPI_Allgather time keeps increasing, thus becoming bottleneck when moved to large machines; even communication time may exceed computation time for simulating large neuronal network models. Limitation on 2–32 processors is depicted in Table 1. Experiments were done on Parscalebush model while increasing number of processors and keeping tstop constant (5000 ms) to analyze the limitation, as illustrated in Figure 4.

## 5. Proposed Method

Collective communication is significant and is adopted in NEURON for communication between processors, but two-sided communication using MPI_Allgather makes implementation for optimization possible. MPI_Allgather is enhanced to RMA_Allgather one-sided communication using recursive doubling for efficient spikes exchange between processors. One-sided communication requires only one processor for communication, thus ensuring that both sender and receiver are not bounded to each other during whole communication process, enabling efficient communication. To minimize the number of steps for efficient spikes exchange recursive doubling mechanism was implemented which reduced the number of steps for exchange, thus ensuring message exchange in *O*(log⁡*p*) steps, where *n* is number of processors. This paper is based on limited processor version of the recursive doubling algorithm for points of multifaceted sharing between multiple processors using time interval for one-sided communication. The following are algorithms for RMA_Allgather and target calculation that were implemented and tested in NEURON.

**Figure 5:**
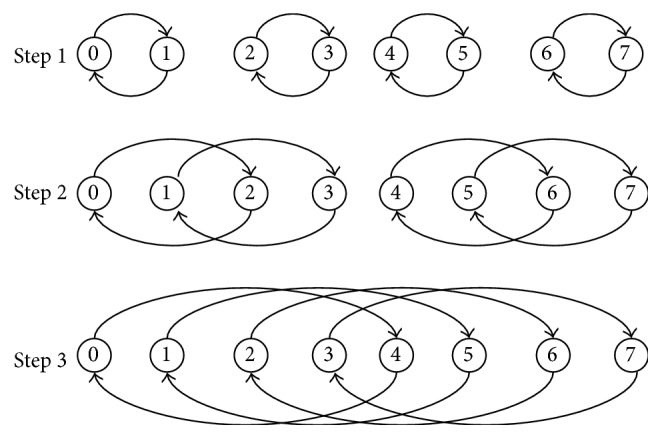
Logical view of RMA_Allgather based on recursive doubling algorithm.

Calculation of target by every processor in each step lays foundation for appropriate communication. In the first step every processor communicates with other processors which is distance 1 apart and as the step increases their distance doubles as in Figure 5.  Algorithm 1 ensures the appropriate target calculation, as it is necessary because in each step target varies according to processor ID and step.

The recursive doubling algorithm is adopted to resolve communication bottleneck on parallel machines with *p* processors in *O*(log⁡*p*) parallel arithmetic steps. In order to allow other processors to write remotely, the processor exposes its memory, and RMA enables the processors to access data and communicate without requiring other processors to be part of communication process. This enables processors to concurrently carry out the communication process.

RMA_Allgather is carried out instead of MPI_Allgather and thus decouples data communication from system synchronization. It is nonblocking approach which allows processes to communicate concurrently without waiting for other processors to synchronize. The whole process is completed efficiently in *O*(log⁡*p*) steps where *n* is number of processors.  Algorithm 2 also enlightens the calculation of exact origin address for message to be communicated which is necessary as size of message to be communicated increases in each step, and after first step each processor has to send its own data along with the spikes information obtained in previous steps. After origin and appropriate target calculation, MPI_Win_Fence is used to synchronize the processors in each step, and every processor uses MPI_Put for writing spikes data on window of remote process; this reduces extra immediate buffering and dual synchronization overhead required in two-sided communication. Thus, combination of both RMA and recursive doubling when applied results in optimization of NEURON for simulation of large neuronal network models.

## 6. Experimental Results

This section will demonstrate the impact of proposed RMA_Allgather method through several experiments performed after implementation of the algorithms in NEURON on a 4-node HP BL460c cluster placed at Kadir Has University. The SMP cluster has 2 × 2.66 GHz Intel Xeon Quad Core CPUs and RAM of 24 GB and has 8 processing cores per node running Linux 2.6.18 connected with 20 Gbps InfiniBand. The experiment is performed 5 times for obtaining each result and the results are average of 5 runs as shown in Tables 2 and 3.

Simulation tests were conducted on two published neuronal network models, Parscalebush and Parbulbnet [7, 8], exhibiting different spike patterns along with implementation of RMA_Allgather with recursive doubling mechanism. To examine the resulting performance of implementation on models, they were examined in diverse environment. The results were calculated for Parbulbnet model along with varying number of processors and cells while numbers of cells were kept constant; see Table 2. For Parscalebush tstop and numbers of processors were varied while tstop was kept constant to obtain the comparative results and analyze the efficiency of the proposed technique; see Table 3.

**Figure 6:**
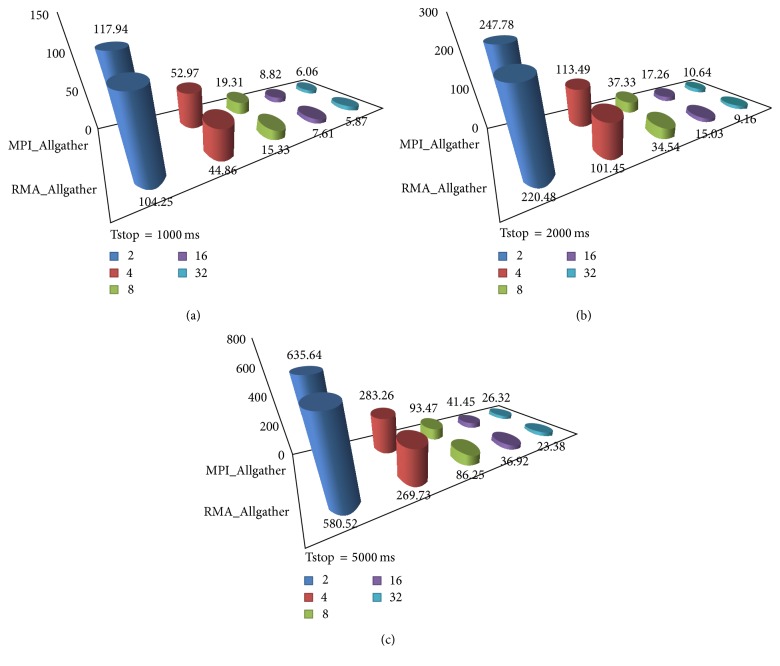
Parbulbnet model runtime comparison between MPI_Allgather and RMA_Allgather with cells = 2525.

**Figure 7:**
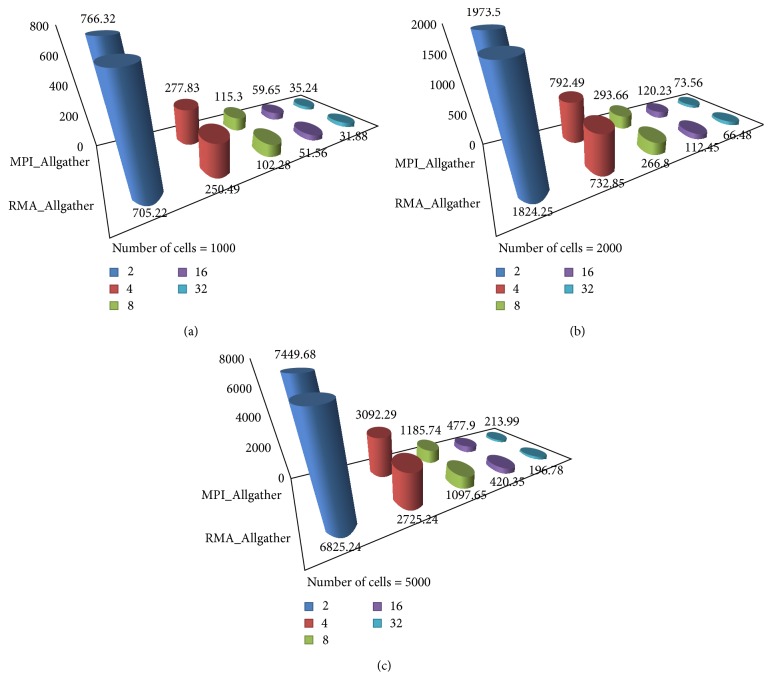
Parscalebush model runtime comparison between MPI_Allgather and RMA_Allgather with tstop = 5000.

It was observed that along with gradual increase in number of processors the size of subnet on single processor becomes smaller and MPI_Allgather becomes source of communication overhead on large number of processors. RMA_Allgather when applied to NEURON provides much better results than existing communication mechanism. Tables 2 and 3 depict that proposed technique is almost 10% more efficient than existing MPI_Allgather adopted in NEURON simulation environment, thus reducing communication time (Figures 6 and 7) and improving the overall efficiency of NEURON.

## 7. Conclusion

Speedup from parallelizing large network models in NEURON is found nearly proportional to number of processors, but spikes exchange time was found inversely affecting runtime along with increasing number of processors. In this paper, an optimization method RMA_Allgather using recursive doubling is applied for exchange of spikes in NEURON simulator that reduces spike exchange time almost 10% as compared to the existing MPI_Allgather method. RMA facilitates advantage of direct memory access to data of remote processor and reduces the synchronization overhead whereas recursive doubling limits the overall communication steps, thus benefiting the performance of NEURON for simulating large neuronal network models. In future we plan to improve remote direct memory access in NEURON by exchanging only the updated spikes to optimize the communication process.

## Figures and Tables

**Algorithm 1:**
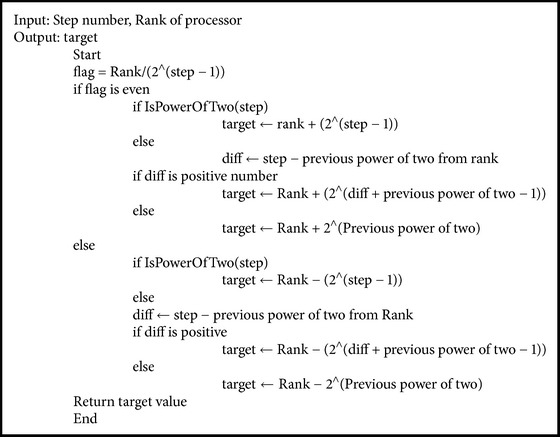
Recursive doubling algorithm for target calculation.

**Algorithm 2:**
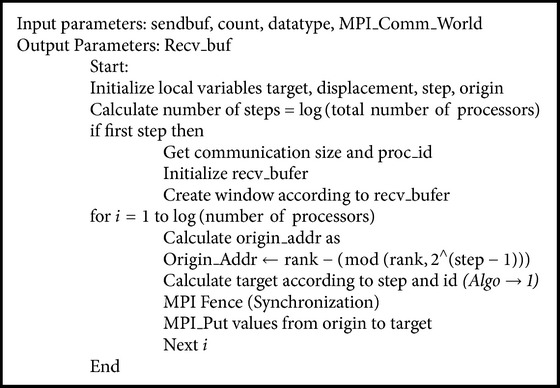
RMA_Allgather for spikes communication between processors.

**Table 1:** Communication versus computation percentage time for Parscalebush model.

Cells	Number of procs.	2	4	8	16	32
500	MPI_Allgather%	0.81	4.98	6.79	6.84	18.02
	Computation time%	99.18	95.01	93.2	93.15	81.97

1000	MPI_Allgather%	2.04	1.69	3.14	7.81	14.26
	Computation time%	97.95	98.3	96.85	92.18	85.73

2000	MPI_Allgather%	2.71	1.18	4.28	3.64	12.88
	Computation time%	97.28	98.81	95.71	96.35	87.11

4000	MPI_Allgather%	1.38	2.8	5.83	4.97	14.29
	Computation time%	98.62	97.19	94.16	95.02	85.7

**Table 2:** Runtime comparison of MPI_Allgather versus RMA_Allgather in Parbulbnet.

Parbulbnet: cells = 2525						
Tstop	1000 ms		2000 ms		5000 ms	
Procs.	MPI	RMA	MPI	RMA	MPI	RMA

2	117.94	104.25	247.78	220.48	635.64	580.52
4	52.97	44.86	113.49	101.45	283.26	269.73
8	19.31	15.33	37.33	34.54	93.47	86.25
16	8.82	7.61	17.26	15.03	41.45	36.92
32	6.06	5.87	10.64	9.16	26.32	23.38

**Table 3:** Runtime comparison of MPI_Allgather versus RMA_Allgather in Parscalebush.

Parscalebush: tstop = 5000 ms						
Cells	1000		2000		5000	
Procs.	MPI	RMA	MPI	RMA	MPI	RMA

2	766.32	705.22	1973.5	1824.25	7449.68	6825.24
4	277.83	250.49	792.49	732.85	3092.29	2725.24
8	115.3	102.28	293.66	266.8	1185.74	1097.65
16	59.65	51.56	120.23	112.45	477.9	420.35
32	35.24	31.88	73.56	66.48	213.99	196.78
